# Analysis of Postdischarge Interventions for Children Treated for Moderate or Severe Wasting, Growth Faltering or Failure, or Edema: A Systematic Review

**DOI:** 10.1001/jamanetworkopen.2023.15077

**Published:** 2023-05-24

**Authors:** Lilia Bliznashka, Susan M. Rattigan, Christopher R. Sudfeld, Sheila Isanaka

**Affiliations:** 1 International Food Policy Research Institute, Washington, DC; 2 Department of Nutrition, Harvard T.H. Chan School of Public Health, Boston, Massachusetts; 3 Department of Global Health and Population, Harvard T.H. Chan School of Public Health, Boston, Massachusetts; 4 Department of Research, Epicentre, Paris, France

## Abstract

**Importance:**

Children treated for acute malnutrition remain at increased risk of relapse, infection, and mortality after programmatic recovery. Global guidelines for the management of acute malnutrition currently provide no recommendations to sustain recovery following treatment discharge.

**Objective:**

To inform guideline development by evaluating the evidence on postdischarge interventions to improve outcomes within 6 months after discharge.

**Evidence Review:**

In this systematic review, 8 databases were searched from inception through December 2021 and included randomized and quasi-experimental studies investigating interventions delivered after discharge from nutritional treatment for children aged 0 to 59 months. Outcomes were relapse, deterioration to severe wasting, readmission, sustained recovery, anthropometry, all-cause mortality, and morbidity within 6 months after discharge. The risk of bias was assessed using Cochrane tools, and the certainty of the evidence was evaluated with the GRADE approach.

**Findings:**

Of 7124 records identified, 8 studies, conducted in 7 countries between 2003 and 2019 with 5965 participants, were included. The study interventions included antibiotic prophylaxis (n = 1), zinc supplementation (n = 1), food supplementation (n = 2), psychosocial stimulation (n = 3), unconditional cash transfers (n = 1), and an integrated biomedical, food supplementation, and malaria prevention package (n = 1). Risk of bias was moderate or high for half the studies. Only unconditional cash transfers were associated with reduced relapse, while the integrated package was associated with improved sustained recovery. Zinc supplementation, food supplementation, psychosocial stimulation, and unconditional cash transfers were associated with improvements in postdischarge anthropometry, while zinc supplementation was associated with reductions in multiple postdischarge morbidities.

**Conclusions and Relevance:**

In this systematic review of postdischarge interventions to reduce relapse and improve other postdischarge outcomes among children treated for acute malnutrition, evidence was limited. Biomedical, cash, and integrated interventions showed promise in improving certain postdischarge outcomes for children treated for moderate or severe acute malnutrition in single studies. Further evidence on the efficacy, effectiveness, and operational feasibility of postdischarge interventions in other contexts is needed to inform global guidance development.

## Key Points

**Question:**

In infants and children treated for growth failure or faltering, moderate or severe wasting, or edema, which postdischarge interventions are helpful in improving outcomes?

**Findings:**

This systematic review, which included 8 intervention studies from 7 countries with 5965 participants, found that biomedical, cash transfer, and integrated interventions may improve certain outcomes in children after treatment for acute malnutrition.

**Meaning:**

Given limited evidence on the efficacy of interventions to improve postdischarge outcomes following nutritional treatment for moderate or severe acute malnutrition, additional research on the effectiveness and operational feasibility is warranted to inform global guidance.

## Teaser

This systematic review evaluates the evidence on postdischarge interventions to improve outcomes among children treated for acute malnutrition within 6 months after discharge.

## Introduction

Wasting (ie, a child being too thin for their height) affects at least 45 million children worldwide^1^ and is associated with increased child morbidity and mortality.^2^ Despite the effectiveness of community-based management of acute malnutrition, children successfully discharged from treatment continue to face increased risks of relapse, infection, and death following nutritional recovery.^3,4,5,6^ A recent systematic review showed that the proportion of children who relapse after successful discharge from nutritional treatment for severe acute malnutrition (SAM) may be as high as 37%.^3^ Other studies indicate postdischarge mortality risk could be as high as 9%.^4,7,8^ An increased burden of morbidities (eg, fever, cough, diarrhea) has also been documented following successful discharge from nutritional treatment.^9,10,11,12^

Despite increased risks of adverse outcomes following nutritional recovery, current World Health Organization (WHO) guidelines provide no or limited recommendations for interventions to improve postdischarge outcomes.^13^ A recent systematic review on postdischarge interventions following hospitalization for complicated SAM concluded that medical and psychosocial interventions showed promise in reducing postdischarge mortality following hospitalization in this specific subset of children with acute malnutrition.^14^ An important knowledge gap exists in our understanding of which interventions are effective to improve postdischarge outcomes in most children treated on an outpatient basis for uncomplicated SAM, moderate acute malnutrition (MAM), growth failure or faltering, or edema. In addition, little is known about the subgroups of children who may benefit from postdischarge interventions. The identification of effective interventions and subgroups of children who may benefit should be used to inform the development of guidelines for postdischarge interventions to prevent relapse and other adverse outcomes after discharge and to improve targeting and the longer-term impact of standard treatment. In 2021, the WHO identified this as a priority area for guideline development and commissioned a systematic review.^15^ The current review aimed to evaluate the evidence on the effectiveness of postdischarge interventions for infants and children treated for moderate or severe wasting, growth failure or faltering, or edema to improve outcomes and to identify subgroups of children who may benefit from postdischarge interventions.

## Methods

This systematic review was conducted following Preferred Reporting Items for Systematic Reviews and Meta-Analysis (PRISMA) reporting guideline^16^ and Cochrane Handbook for Systematic Reviews of Interventions^17^ guidelines. The protocol was registered with Prospero (CRD42022308380).

### Search Strategy and Selection Criteria

We searched PubMed, Cochrane Library, Embase, Web of Science Index Medicus, CINAHL, Latin American and Caribbean Health Sciences Literature (LILACS), e-Library of Evidence for Nutrition Actions (eLENA; WHO), and Index Medicus for the Eastern Mediterranean Region (IMEMR) from inception through December 2021 without language or geographical restrictions. The search strategy was developed using index terms along the following themes: child or infant, wasting, intervention, and discharge (eAppendix 1 in Supplement 1). References of extracted articles were reviewed for additional studies to include.

Peer-reviewed articles were included if they (1) assessed children aged 0 to 59 months treated for complicated or uncomplicated MAM or SAM, growth faltering or failure, or edema; (2) examined any intervention delivered partially or completely after discharge from nutritional treatment, where after discharge was defined as following exit from all phases of nutritional treatment; (3) study design was individually randomized clinical trial (RCT), cluster RCT, quasi-randomized study, controlled before-after study, or interrupted time series; and (4) assessed relapse, deterioration to severe wasting, readmission, anthropometric measures, all-cause mortality, or morbidity up to 6 months after discharge. Outcome definitions are provided in eAppendix 2 in Supplement 1.

### Data Analysis

Two reviewers (L.B. and S.M.R.) independently screened titles, abstracts, and full texts for inclusion. Disagreements were resolved through discussion with a third reviewer (S.I.). Data were extracted and reviewed using a standardized form. The risk of bias was assessed by 2 reviewers (L.B. and S.M.R.) using the Cochrane Risk of Bias 2 tool for RCTs and cluster RCTs^18^ and the Cochrane Risk of Bias in Nonrandomized Studies of Interventions (ROBINS-I) tool for nonrandomized studies,^19^ and the certainty of the evidence using the GRADE approach.^20,21^ Disagreements were resolved through discussion with a third reviewer (S.I.). Authors of the original studies were not contacted for clarification or additional information.

Data were synthesized narratively by study characteristics, intervention characteristics, and outcomes assessed. Included interventions were classified by type based on the hypothesized mechanisms of action: biomedical, food supplementation, psychosocial stimulation, cash transfers, or integrated packages operating through multiple mechanisms. Results were summarized narratively and presented by intervention type. Considering that food availability is a key driver of child wasting and the provision of food supplementation likely affected outcomes, we summarized results on psychosocial stimulation alone in studies providing psychosocial stimulation with and without food supplementation. Meta-analysis was planned when at least 2 studies assessed similar interventions and predefined outcomes. Meta-analysis did not pool experimental and quasi-experimental studies.^17^ Subgroup meta-analysis to examine effect modification by child and study characteristics was planned (eAppendix 3 in Supplement 1) but not conducted as only 1 study reported subgroup analyses.

## Results

We identified 7124 records (Figure). After removing duplicates and screening titles and abstracts, we evaluated 26 full texts, of which 18 were excluded (eTable 1 in Supplement 1). We included 8 studies comprising 5 RCTs,^22,23,24,25,26^ 2 cluster-RCTs,^27,28^ and 1 quasi-experimental study^29^ from 7 countries published between 2003 and 2019 (Table 1). Analytic sample sizes ranged from 80 to 1778 children per study (pooled population, 5965 participants). Six studies enrolled children admitted in hospital,^22,23,24,25,26,29^ 1 in an outpatient therapeutic program setting,^27^ and 1 in a community-based supplementary feeding program setting.^28^ In 4 of the 6 studies that enrolled children admitted in hospital,^22,23,24,29^ there was no outpatient treatment at the time of the studies and discharge from inpatient treatment constituted complete exit from nutritional treatment. In the remaining 2 studies that enrolled children admitted in hospital,^25,26^ children were discharged from inpatient treatment to outpatient treatment for continued follow-up. Study populations included children treated for SAM (n = 6),^22,24,25,26,27,29^ MAM (n = 1),^28^ and protein energy malnutrition (n = 1).^23^ The review identified no studies of children treated for growth faltering, growth failure, or edema.

**Figure.**
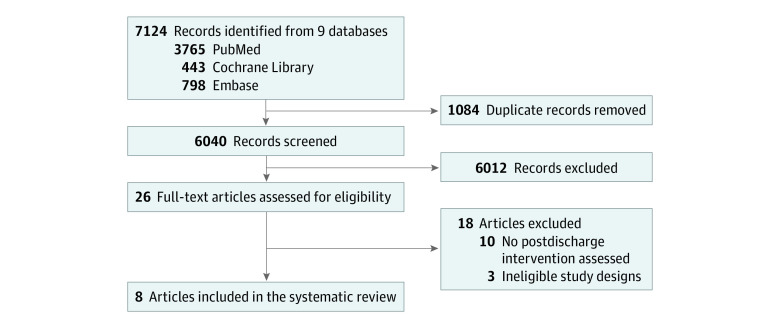
Study Flow Diagram

**Table 1.** Characteristics of the 8 Studies Included in the Systematic Review

Source	Country, setting, and study design	Length of follow-up	Summary of population assessed	Sample size	Summary of intervention	Summary of comparator	Intervention duration	Outcomes assessed	Summary of findings	Risk of bias
Abessa et al,^25^ 2019	Ethiopia, hospital, RCT	6 mo	Children aged 6-60 mo with uncomplicated SAM.	339: 169 intervention and 170 control	Psychosocial support stimulation: 2 phases of inpatient and outpatient of play-based psychosocial stimulation.	Biomedical and dietary treatment	Up to 6 mo after discharge from inpatient treatment	Anthropometric measures	No association with improved anthropometric measures by end of follow-up	Some concerns
Berkley et al,^26^ 2016	Kenya, hospital, RCT	12 mo	Children aged 60 d to 59 mo diagnosed with SAM.	1778: 887 intervention and 891 control	Biomedical: 6 mo of daily oral co-trimoxazole prophylaxis.	Placebo	6 mo from discharge from inpatient treatment.	Relapse to SAM, sustained recovery, anthropometric measure, all-cause mortality, morbidity or recovery from morbidity	No association with reduced mortality or adverse events or improved anthropometric outcomes	Low
Chauhan et al,^22^ 2019	India, hospital, RCT	1.5 mo	Children aged 6 mo to 5 y with SAM	80: 40 intervention and 40 control	Food supplementation: nonmilk based LTF provided at discharge and 3 follow-ups every 2 weeks.	Advice on home-based diet, no LTF received	6 weeks starting at discharge from inpatient treatment	Anthropometric measures	The intervention was associated with improved weight gain over 6 weeks of follow-up and anthropometric outcomes; a greater number of children in the intervention group were labeled as cured.	Some concerns
Grellety et al,^27^ 2017	Democratic Republic of the Congo, OTP, cluster RCT	6 mo	Children aged 6-59 mo in outpatient treatment for uncomplicated SAM	1481: 734 intervention and 747 control	Cash transfer: all caregivers with ≥1 children with SAM received an unconditional cash transfer of US $40 per month during treatment and follow-up for a total of 6 mo.	Standard of care.	A total of 6 mos starting at admission to outpatient treatment and continuing after discharge	Relapse, sustained recovery, anthropometric measures	Intervention associated with decreased risk of MAM and SAM relapse and increased weight and MUAC gain. Changes in WAZ, WHZ, BMIZ, MUACZ, and MUAC-for-height *z *score were all positive in the intervention group.	Low
Makonnen et al,^23^ 2003	Lesotho, hospital, RCT	3 mo	Children aged 6 mo to 5 y with signs of protein energy malnutrition based on the 1999 Wellcome Classification or signs and symptoms of kwashiorkor	300: 150 intervention and 150 control	Biomedical: daily dose of 10 mg of zinc was administered from the first day of admission to 90 d postdischarge	Placebo	Starting at admission to inpatient treatment and continuing until 90 d after discharge from inpatient treatment.	Anthropometric measures, all-cause mortality, morbidity or recovery from morbidity.	Intervention associated with reduced morbidity at 30, 60 and 90 d after discharge as well as improved WAZ at 30, 60 and 90 d after discharge and MUAC<5% at 60 and 90 d after discharge.	Low
Nahar et al,^29^ 2009	Bangladesh, hospital, time-lagged controlled study	6 mo	Children aged 6 to 24 mo hospitalized with SAM	97: 54 intervention and 43 control	Psychosocial stimulation: 18 total sessions in hospital, at home, and the hospital for follow-up visits.	Routine nutritional and health care and health and nutrition education	From admission to 6 mo after discharge from inpatient treatment	Anthropometric measures	Intervention associated with improved WAZ at 6 mo after discharge.	Serious
Nahar et al,^24^ 2012	Bangladesh, hospital, RCT	6 mo	Children aged 6-24 mo hospitalized with severe underweight without acute infections.	507: 102 psychosocial stimulation, 101 food supplementation, 103 psychosocial stimulation and food supplementation, 99 clinic control, 102 hospital control	Psychosocial stimulation: individual play sessions and parental education; food supplementation: food packets for 3 mo.	Fortnightly follow-up at clinic or hospital with growth monitoring, health education, and micronutrient supplementation	6 mo	Anthropometric measures	No intervention effect on anthropometric outcomes after 6 mo of intervention. Any psychosocial stimulation improved WAZ compared with no stimulation at 6 mo.	Low
Stobaugh et al,^28^ 2017	Malawi, community-based supplementary feeding program, cluster RCT	12 mo	Children aged 6-62 mos discharged as recovered from community-based treatment for MAM.	1383: 769 intervention and 718 control	Intervention package consisting of the following interventions: food supplementation, biomedical support, and malaria prevention	Standard of care and routine nutrition and health counselling	Up to 1 y after discharge from outpatient treatment	MAM relapse, deterioration to severe wasting among children recovered from moderate wasting, sustained recovery, all-cause mortality	The intervention was associated with improved sustained recovery at 1, 3, and 6 mo after discharge, but had no effect on sustained recovery at 12 mo (primary outcome).	High

Abbreviations: BMIZ, body mass index *z *score; LTF, local therapeutic food; MAM, moderate acute malnutrition; MUAC, mid–upper arm circumference; MUACZ, mid–upper arm circumference *z *score; OTP, outpatient therapeutic program; RCT, randomized clinical trial; SAM, severe acute malnutrition; WAZ, weight-for-age *z *score; WHZ, weight-for-height/length *z *score.

Evaluated interventions included biomedical interventions (n = 2),^23,26^ food supplementation (n = 1),^22^ psychosocial stimulation (n = 2),^25,29^ food supplementation and/or psychosocial stimulation (n = 1),^24^ unconditional cash transfers (n = 1),^27^ and an integrated package providing biomedical intervention, food supplementation, and malaria prevention (n = 1).^28^ Mean duration of postdischarge follow-up was 9.3 months (range: 1.5 months^22^ to 12 months^26,28^). Analyzed outcomes are summarized in Table 2. Risk of bias was low for 4 of the included studies,^23,24,26,27^ moderate for 2,^22,25^ and high for 2,^28,29^ with bias arising from the randomization process, potential selection of the reported results, and missing data (Table 1; eTable 2 and eFigures 1-3 in Supplement 1).

**Table 2.** Outcomes Analyzed by the 8 Studies Included in the Systematic Review

Outcome	Studies reporting outcome, No. (%)	Specific studies reporting each outcome							
		Abessa et al,^25^ 2019	Berkley et al,^26^ 2016	Chauhan et al,^22^ 2019	Grellety et al,^27^ 2017	Makonnen et al,^23^ 2003	Nahar et al,^29^ 2009	Nahar et al,^24^ 2012	Stobaugh et al,^28^ 2017
Relapse									
Relapse to moderate acute malnutrition	2 (25)	No	No	No	Yes	No	No	No	Yes
Relapse to severe acute malnutrition	2 (25)	No	Yes	No	Yes	No	No	No	No
Deterioration to severe wasting among children recovered from moderate wasting	1 (13)	No	No	No	No	No	No	No	Yes
Readmission	0	No	No	No	No	No	No	No	No
Sustained recovery	0	No	Yes	No	No	No	No	No	No
Sustained recovery	3 (38)	No	Yes	No	Yes	No	No	No	Yes
Anthropometric measures									
Body mass index *z *score	1 (13)	No	No	No	Yes	No	No	No	No
Height-for-age *z *score	4 (50)	Yes	Yes	No	Yes	No	No	Yes	No
Head circumference-for-age *z *score	1 (13)	No	Yes	No	No	No	No	No	No
Height	1 (13)	No	No	No	Yes	No	No	No	No
Mid–upper arm circumference	4 (50)	No	Yes	Yes	Yes	Yes	No	No	No
Mid–upper arm circumference for height *z *score	1 (13)	No	No	No	Yes	No	No	No	No
Mid–upper arm circumference *z *score	2 (25)	Yes	No	No	Yes	No	No	No	No
Weight-for-age *z *score	6 (75)	Yes	Yes	No	Yes	Yes	Yes	Yes	No
Weight	1 (13)	No	No	No	Yes	No	No	No	No
Weight gain	1 (13)	No	No	Yes	No	No	No	No	No
Weight-for-height *z *score	5 (63)	Yes	Yes	Yes	Yes	No	No	Yes	No
All-cause mortality	2 (25)	No	Yes	No	No	No	No	No	Yes
Morbidity or recovery from co-morbidity									
Incidence of acute respiratory infection	1 (13)	No	No	No	No	Yes	No	No	No
Incidence of diarrhea	2 (25)	No	Yes	No	No	Yes	No	No	No
Incidence of fever	1 (13)	No	No	No	No	Yes	No	No	No
Incidence of lower respiratory tract infection	1 (13)	No	Yes	No	No	No	No	No	No
Incidence of malaria	1 (13)	No	Yes	No	No	No	No	No	No
Incidence of edema	1 (13)	No	No	No	No	Yes	No	No	No
Incidence of outpatient clinical episodes	1 (13)	No	Yes	No	No	No	No	No	No
Incidence of pallor	1 (13)	No	No	No	No	Yes	No	No	No
Incidence of pneumonia	1 (13)	No	Yes	No	No	No	No	No	No
Incidence of skin infection	2 (25)	No	Yes	No	No	Yes	No	No	No
Incidence of upper respiratory tract infection	1 (13)	No	Yes	No	No	No	No	No	No
Incidence of urinary tract infection	1 (13)	No	Yes	No	No	No	No	No	No
Incidence of vomiting	1 (13)	No	No	No	No	Yes	No	No	No

One study assessed daily antibiotic prophylaxis with oral co-trimoxazole compared with placebo for 6 months starting at discharge from inpatient treatment and continuing during and after outpatient treatment.^26^ Estimates for associations after discharge from nutritional treatment could not be extracted, as the authors did not report postdischarge estimates from all nutritional treatment (only postdischarge estimates of inpatient treatment, which included follow-up during subsequent outpatient treatment and postoutpatient discharge). Associations at 6 months and 12 months after discharge from inpatient treatment for antibiotic prophylaxis with oral co-trimoxazole are summarized in eAppendix 4 and eTable 3 in Supplement 1.

Daily supplementation with 10 mg of zinc starting at admission to inpatient treatment and continuing until 90 days after discharge was associated with higher mid–upper arm circumference (MUAC) and weight-for-age *z *score (WAZ) at 90 days after discharge; lower prevalence of diarrhea, skin infections, vomiting, fever, acute respiratory infection, and pallor at 30, 60, and 90 days after discharge; and lower prevalence of edema at 30 and 60 days after discharge.^23^ The certainty of the evidence was downgraded to moderate for all outcomes due to imprecision, ie, small sample size, few events, and wide confidence intervals (eTable 4 in Supplement 1).

Two studies assessed food supplementation.^22,24^ Daily food supplementation (150 kcal/d) for 3 months starting at discharge from inpatient SAM treatment was not associated with postdischarge anthropometric *z* scores.^24^ Supplementation with a nonmilk based local therapeutic food (833 kcal/d) for 6 weeks starting at discharge from inpatient SAM treatment was associated with lower proportion of children with low MUAC or low weight-for-height *z *score (WHZ) at 6 weeks after discharge.^22^ The certainty of the evidence was downgraded to moderate for anthropometric *z* scores due to imprecision (small sample size and wide confidence intervals) and to low for weight gain and MUAC due to high risk of bias and imprecision (small sample size and no measure of uncertainty reported) (eTable 5 in Supplement 1).

Of the 3 studies that assessed psychosocial stimulation, 2 provided psychosocial stimulation alone^25,29^ and 1 provided psychosocial stimulation with and without food supplementation.^24^ Meta-analysis of the 2 studies that provided psychosocial stimulation alone was not conducted, as 1 study was quasi-experimental.^29^ Estimates for associations after discharge from nutritional treatment could not be extracted from 1 study,^25^ as the authors did not provide postdischarge estimates from all nutritional treatment (only postdischarge estimates of inpatient treatment, which included follow-up during subsequent outpatient treatment and post–outpatient discharge). Associations at 6 months after discharge from inpatient treatment are summarized in eAppendix 5 and eTable 6 in Supplement 1.

Psychosocial stimulation delivered to children aged 6 to 24 months hospitalized with severe underweight (WAZ <−3 SD) through biweekly 1-hour sessions for 6 months was not associated with anthropometric *z* scores at 6 months after discharge.^24^ In contrast, psychosocial stimulation for hospitalized, severely underweight children aged 6 to 24 months starting at admission and continuing for 6 months after discharge (delivered through individual and group sessions in hospital and home visits after discharge) was associated with higher mean (SD) WAZ vs children who did not receive the intervention after 6 months of follow-up: −3.1 (0.9) vs −3.6 (1.2); *P* = .03.^29^ The certainty of evidence on the effect of psychosocial stimulation was very low for all anthropometric *z* scores due to downgrades for risk of bias, inconsistency, and imprecision (eTable 6 and eTable 7 in Supplement 1).

Unconditional cash transfers among children aged 6 to 59 months of age in an outpatient SAM treatment program (US $40/mo for 6 months starting at admission) was associated with lower risk of MAM relapse (hazard ratio [HR], 0.21; 95% CI, 0.11-0.41) and SAM relapse (HR, 0.30; 95% CI, 0.16-0.58) and positive changes in weight, MUAC, and weight-related anthropometric *z* scores.^27^ The certainty of the evidence was downgraded to moderate due to indirectness, related to limited generalizability of the study (eTable 8 in Supplement 1).

One study assessed an integrated package providing biomedical support (a single dose of albendazole and zinc supplementation for 14 days), food supplementation (40 g/d of lipid-based nutrient supplement, providing 200 kcal and 1 recommended daily allowance of micronutrients for 8 weeks), and malaria prevention (provision of an insecticide-treated bed net and a monthly dose of 25 mg/kg of sulfadoxine-pyrimethamine for malaria chemoprophylaxis during the peak of the rainy season) starting at discharge from MAM treatment.^28^ The integrated package was not associated with MAM relapse, deterioration to severe wasting, or all-cause mortality after 1, 3, or 6 months of follow-up. The intervention was associated with higher prevalence of sustained recovery after 1 month (intervention vs control: 78% vs 74%; *P* = .04), 3 months (intervention vs control: 69% vs 63%; *P* = .02), and 6 months (intervention vs control: 64% vs 59%; *P* = .04).^28^ The certainty of the evidence was downgraded 2 levels for all outcomes due to risk of bias and imprecision (eTable 9 in Supplement 1).

## Discussion

In this systematic review aiming to analyze postdischarge interventions for children successfully treated for complicated or uncomplicated MAM or SAM, growth failure or faltering, or edema, we included 8 studies conducted from 2003 to 2019 that evaluated biomedical, food supplementation, psychosocial stimulation, cash transfer, and integrated interventions delivered after discharge from nutritional treatment. One zinc supplementation trial was associated with improved MUAC and WAZ and reduced morbidities within 90 days after discharge.^23^ One of the 2 food supplementation interventions, which provided a nonmilk-based local therapeutic food, was associated with improved postdischarge weight gain, MUAC, and WHZ.^22^ Psychosocial stimulation was associated with improved WAZ at 6 months after discharge in one quasi-experimental study.^29^ One unconditional cash transfer intervention was associated with reduced MAM and SAM relapse and improved anthropometric measures.^27^ The integrated biomedical, nutrition, and malaria package following MAM treatment showed some evidence of benefits on sustained recovery and protection from deterioration to severe wasting within 6 months of discharge.^28^ None of the included interventions reduced the risk of all-cause mortality. The review did not identify studies among children treated for growth failure or faltering or edema.

Infections are a main cause of mortality during SAM recovery.^30^ Growing evidence demonstrates elevated morbidity in the postdischarge period despite anthropometric recovery.^9,10,11,12,31^ Zinc supplementation is effective for reducing diarrhea risk in children,^32,33^ including in those recovering from protein energy malnutrition^34^ and particularly in areas with high prevalence of malnutrition and zinc deficiency.^35^ Preventive zinc supplementation might also reduce the incidence of pneumonia.^36^ The postdischarge zinc supplementation intervention included in this review was associated with reduced risk of multiple morbidities, including diarrhea and acute respiratory infection, and improvement in anthropometry after discharge,^23^ suggesting zinc supplementation may be an effective strategy to reduce the risk of other morbidities beyond diarrhea. While having a low risk of bias, this was a single, older study that applied a now outdated nutrition treatment protocol and discharge criterion (ie, recovery defined at WHZ >80% of expected weight). These findings should be replicated in other contemporary settings while exploring questions related to the effective dose and duration of zinc supplementation.

Current WHO guidelines for inpatient SAM treatment recommend child play activities during treatment to continue after program discharge.^37^ Our review included 2 RCTs^24,25^ and 1 quasi-experimental study^29^ of psychosocial stimulation interventions. The psychosocial stimulation interventions were of varying intensity and duration and overall showed small to no association with anthropometric measures after discharge from all nutritional treatment. When follow-up from discharge from inpatient treatment and from all nutritional treatment were pooled in an ad hoc meta-analysis, the intervention was associated with WAZ and HAZ (eTable 7 in Supplement 1). More research could be beneficial to assess the effectiveness and cost-effectiveness of combining psychosocial stimulation with other interventions to improve postdischarge nutritional and child development outcomes. Additional work is also needed to understand and minimize any potential increased burden for caregivers and health workers’ from attending or delivering psychosocial stimulation sessions.

We found limited evidence of benefit of direct food supplementation. One randomized study showed no benefit of food supplementation (150 kcal/d for 3 months) on postdischarge anthropometric measures.^24^ Another indicated some benefits of food supplementation (833 kcal/d for 6 weeks) on postdischarge anthropometric measures.^22^ However, the latter was a relatively small study at serious risk of bias. International organizations, like the World Food Programme, have increasingly been shifting toward the provision of cash in emergency and humanitarian settings, as studies indicate that cash distribution can be more efficient than direct food aid, allowing more people to be reached with cash at no extra cost.^38,39^ A single study showed that unconditional cash transfers was associated with reduced MAM and SAM relapse and improved anthropometric measures within 6 months from admission.^27^ The unconditional cash transfer was hypothesized to improve child outcomes by several mechanisms, including reducing sharing of therapeutic foods during nutritional treatment, increasing household food accessibility and consumption, reducing morbidities by providing money for health care, and increasing income generating opportunities by serving as investment capital.^27^ However, evidence on the effectiveness of a cash intervention in one setting may not directly generalize to other contexts. The size of the cash transfer in the included study was relatively large, corresponding to 70% of the monthly household income of very poor households in this setting,^27^ which likely contributed to its success in improving a range of nutritional outcomes. More research is needed on the optimal amount, duration, and any potential unintended behavioral and market consequences of postdischarge cash transfers in different settings.

Our findings also showed that an integrated package of interventions may be a promising strategy to improve certain postdischarge outcomes in children treated for MAM. The package included food and zinc supplementation, antibiotic prophylaxis, and malaria prevention and was associated with sustained recovery within 6 months after discharge, but effect sizes were overall small. The integrated package had no association with relapse, deterioration to severe wasting, or all-cause mortality,^28^ which may be due to its short duration (longest intervention was food supplementation provided for 8 weeks). Although the intervention improved sustained recovery within 6 months after discharge, the authors reported that intervention effect sizes were no longer significant after 12 months of follow-up. More work is needed to understand long-term outcomes and why recovery was not sustained over the longer period of follow-up and to determine which intervention components of the package may be beneficial. Replication in other settings is also warranted to assess the feasibility and effectiveness of packaging these same interventions in different contexts.

### Limitations

This study has limitations. First, included studies assessed various interventions that differed in intensity and duration. Some interventions started at discharge from nutritional treatment and continued for a fixed duration after discharge, while others started during treatment and continued for a varying duration after discharge, which led to variable duration of exposure. It is possible that the duration of some interventions might have been insufficient to meaningfully improve postdischarge outcomes. Second, results from some studies could not be summarized when authors reported postdischarge estimates from inpatient treatment and not postdischarge estimates from all nutritional treatment. Future studies should provide more precise documentation of all phases of nutritional treatment and the number of outcomes by phase. Third, outcomes were measured at different points, with some measured only shortly after discharge while others measured after 6 months of follow-up. The length of follow-up in some studies might have been insufficient to detect changes in the examined outcomes. Fourth, author-reported definitions of outcomes like relapse and sustained recovery varied across studies, making it difficult to draw conclusions. Fifth, only 1 study reported subgroup analysis, and we were therefore unable to identify specific groups of children who may benefit more from postdischarge interventions. Given these limitations, our findings should be interpreted with caution until more evidence on postdischarge interventions is generated.

## Conclusions

This systematic review included 8 studies of postdischarge interventions among children being treated for acute malnutrition. Despite the known risk for postdischarge mortality and poor clinical and nutritional outcomes for children successfully treated in acute malnutrition programs, this review confirms a paucity of evidence on the effectiveness of postdischarge interventions at present. The limited existing evidence suggests that biomedical, cash transfer, and integrated interventions may be promising strategies to improve outcomes following treatment for moderate or severe wasting. Consideration should be extended to assess associations with all-cause mortality and readmission following discharge. Rigorous evidence on the efficacy, effectiveness, and programmatic feasibility of postdischarge interventions is urgently needed to inform global policy and program implementation.

## Notes

Supplement 1. **eAppendix 1.** Search Terms Used in PubMed **eAppendix 2.** Outcome Definitions **eAppendix 3.** Prespecified Subgroups for Subgroup Analyses **eAppendix 4.** Effect of Antibiotic Prophylaxis With Co-trimoxazole **eAppendix 5.** Effect of Psychosocial Stimulation Postdischarge From Inpatient Treatment **eFigure 1.** Risk of Bias for Individually Randomized Clinical Studies Included in the Review **eFigure 2.** Risk of Bias for Cluster Randomized Clinical Studies Included in the Review **eFigure 3.** Risk of Bias for Observational Studies Included in the Review **eTable 1.** Excluded Records During Full-Text Review and Reasons for Exclusion **eTable 2. **Detailed Summary of Included Studies **eTable 3.** GRADE Evidence Profile for the Effect of Daily Oral Co-trimoxazole Prophylaxis Compared With Routine Care (at 12 Months After Discharge From Inpatient Treatment) **eTable 4.** GRADE Evidence Profile for the Effect of Zinc Supplementation Compared With Placebo **eTable 5.** GRADE Evidence Profile for the Effect of Food Supplementation Compared With Routine Care **eTable 6.** GRADE Evidence Profile for the Effect of Psychosocial Stimulation Compared With Routine Care (at 6 Months After Discharge From All Nutritional Treatment) **eTable 7.** GRADE Evidence Profile for the Effect of Psychosocial Stimulation Compared With Routine Care (at 6 Months After Discharge From Inpatient Treatment) **eTable 8.** GRADE Evidence Profile for the Effect of Unconditional Cash Transfers Compared With Routine Care **eTable 9.** GRADE Evidence Profile for the Effect of an Integrated Package of Medical Care, Food Supplementation, and Malaria Prevention Compared With Routine Care

Supplement 2.
Data Sharing Statement
