# Supplementary material for: Analysis of Postdischarge Interventions for Children Treated for Moderate or Severe Wasting, Growth Faltering or Failure, or Edema: A Systematic Review
Source: JAMA Netw Open. 2023 May 24;6(5):e2315077. doi: 10.1001/jamanetworkopen.2023.15077 (PMC10209742; doi:10.1001/jamanetworkopen.2023.15077)
Supplement: Supplement 1. — eAppendix 1. Search Terms Used in PubMed eAppendix 2. Outcome Definitions eAppendix 3. Prespecified Subgroups for Subgroup Analyses eAppendix 4. Effect of Antibiotic Prophylaxis With Co-trimoxazole eAppendix 5. Effect of Psychosocial Stimulation Postdischarge From Inpatient Treatment eFigure 1. Risk of Bias for Individually Randomized Clinical Studies Included in the Review eFigure 2. Risk of Bias for Cluster Randomized Clinical Studies Included in the Review eFigure 3. Risk of Bias for Observational Studies Included in the Review eTable 1. Excluded Records During Full-Text Review and Reasons for Exclusion eTable 2. Detailed Summary of Included Studies eTable 3. GRADE Evidence Profile for the Effect of Daily Oral Co-trimoxazole Prophylaxis Compared With Routine Care (at 12 Months After Discharge From Inpatient Treatment) eTable 4. GRADE Evidence Profile for the Effect of Zinc Supplementation Compared With Placebo eTable 5. GRADE Evidence Profile for the Effect of Food Supplementation Compared With Routine Care eTable 6. GRADE Evidence Profile for the Effect of Psychosocial Stimulation Compared With Routine Care (at 6 Months After Discharge From All Nutritional Treatment) eTable 7. GRADE Evidence Profile for the Effect of Psychosocial Stimulation Compared With Routine Care (at 6 Months After Discharge From Inpatient Treatment) eTable 8. GRADE Evidence Profile for the Effect of Unconditional Cash Transfers Compared With Routine Care eTable 9. GRADE Evidence Profile for the Effect of an Integrated Package of Medical Care, Food Supplementation, and Malaria Prevention Compared With Routine Care [file jamanetwopen-e2315077-s001.pdf]

Supplemental Online Content

Bliznashka L, Rattigan SM, Sudfeld CR, Isanaka S. Analysis of postdischarge interventions for children treated for moderate or severe wasting, growth faltering or failure, or edema: a systematic review. *JAMA Netw Open*. 2023;6(5):e2315077. doi:10.1001/jamanetworkopen.2023.15077

eAppendix 1. Search Terms Used in PubMed

eAppendix 2. Outcome Definitions

eAppendix 3. Prespecified Subgroups for Subgroup Analyses

eAppendix 4. Effect of Antibiotic Prophylaxis With Co-trimoxazole

eAppendix 5. Effect of Psychosocial Stimulation Postdischarge From Inpatient Treatment

eFigure 1. Risk of Bias for Individually Randomized Clinical Studies Included in the Review

eFigure 2. Risk of Bias for Cluster Randomized Clinical Studies Included in the Review

eFigure 3. Risk of Bias for Observational Studies Included in the Review

eTable 1. Excluded Records During Full-Text Review and Reasons for Exclusion

eTable 2. Detailed Summary of Included Studies

eTable 3. GRADE Evidence Profile for the Effect of Daily Oral Co-trimoxazole Prophylaxis Compared With Routine Care (at 12 Months After Discharge From Inpatient Treatment)

eTable 4. GRADE Evidence Profile for the Effect of Zinc Supplementation Compared With Placebo

eTable 5. GRADE Evidence Profile for the Effect of Food Supplementation Compared With Routine Care

eTable 6. GRADE Evidence Profile for the Effect of Psychosocial Stimulation Compared With Routine Care (at 6 Months After Discharge From All Nutritional Treatment)

eTable 7. GRADE Evidence Profile for the Effect of Psychosocial Stimulation Compared With Routine Care (at 6 Months After Discharge From Inpatient Treatment)

eTable 8. GRADE Evidence Profile for the Effect of Unconditional Cash Transfers Compared With Routine Care

eTable 9. GRADE Evidence Profile for the Effect of an Integrated Package of Medical Care, Food Supplementation, and Malaria Prevention Compared With Routine Care

This supplemental material has been provided by the authors to give readers additional information about their work.

© 2023 Bliznashka L et al. *JAMA Network Open.*

eAppendix 1. Search Terms Used in PubMed

("Child"[Mesh] OR "Child, Preschool"[Mesh] OR "child*"[tiab] OR "Infant"[Mesh] OR "infant*"[tiab] OR "p?diatric*"[tiab] OR "toddler*"[tiab] OR "Infant, Newborn"[Mesh] OR "newborn*"[tiab] OR "neonat*"[tiab] OR "early life"[tiab] OR "infants under 6 months" [tiab])

AND

("pre?term"[tiab] OR "pre?mature"[tiab] OR "Infant, Premature"[Mesh] OR "Infant, Low Birth Weight"[Mesh] OR "low birth?weight"[tiab] "LBW"[tiab] OR "Infant, Very Low Birth Weight"[Mesh] OR "very low birth?weight"[tiab] OR "VLBW" [tiab] OR "Infant, Small for Gestational Age"[Mesh] OR "small for gestational age"[tiab] OR "Failure to Thrive"[Mesh] OR "FTT"[tiab] OR "growth failure" [tiab] OR "growth faltering" [tiab] OR "Growth Disorders" [Mesh])

AND

("acute malnutrition"[tiab] OR "Severe Acute Malnutrition"[Mesh] OR "SAM"[tiab] OR "severe acute malnutrition"[tiab] OR "severe malnutrition"[tiab] OR "moderate acute malnutrition"[tiab] OR "severe wasting"[tiab] OR "severely wasted"[tiab] OR "undernourished"[tiab] "severely undernourished"[tiab] OR "severe undernutrition"[tiab] OR "Protein-Energy Malnutrition"[Mesh] OR "PEM"[tiab] OR "malnourish*"[tiab] OR "malnutrition"[tiab] OR "Malnutrition"[Mesh] OR "undernutrition"[tiab] OR "stunt*"[tiab] OR "wast*"[tiab] OR "weight for height"[tiab] OR "weight-for-height"[tiab] OR "weight for length"[tiab] OR "weight-for-length"[tiab] OR "weight for age"[tiab] OR "weight-for-age"[tiab] OR "mid-upper arm circumference"[tiab] OR "mid upper arm circumference"[tiab] OR "MUAC"[tiab] OR "starv*"[tiab] OR "marasmus"[tiab] OR "Kwashiorkor"[Mesh] OR "?edema"[tiab] OR "Edema"[Mesh] OR "?edematous"[tiab] OR "bilateral ?edema"[tiab] OR "bilateral pitting ?edema"[tiab] OR)

AND

("post-discharge"[tiab] OR "discharge"[tiab] OR "after discharge"[tiab] OR "after recovery"[tiab] OR "after cured"[tiab] OR "post-SAM"[tiab] OR "after exit"[tiab] OR "after treatment"[tiab])

AND

("intervention*"[tiab] OR "trial*"[tiab] OR "therap*"[tiab] OR "treat*"[tiab] OR "program*"[tiab] OR "Program"[Mesh] OR "RCT"[All fields])

© 2023 Bliznashka L et al. *JAMA Network Open.*

eAppendix 2. Outcome Definitions

- Relapse: Proportion of children discharged as cured who relapsed as defined by individual authors (caregiver-reported, program-reported, or calculated based on direct anthropometric assessment) within 6 months post-discharge from treatment for growth faltering/failure, complicated or uncomplicated moderate or severe wasting, or oedema.
- Deterioration to severe wasting (weight-for-height Z-score <-3) among children recovered from moderate wasting (weight-for-height Z-score between ≥-2 and <-3): Proportion of children meeting criteria for anthropometric admission for inpatient or outpatient treatment for growth failure/faltering, complicated or uncomplicated severe wasting, or oedema within 6 months after discharge from treatment for moderate wasting.
- Re-admission: Proportion of children re-admitted for inpatient or outpatient treatment for growth failure/faltering, complicated or uncomplicated moderate or severe wasting, or oedema up to 6 months post-discharge as defined by individual authors.
- Sustained recovery: Proportion of children with sustained nutritional recovery up to 6 months post-discharge from treatment for growth faltering/failure, complicated or uncomplicated moderate or severe wasting, or oedema as defined by individual authors (caregiver-reported, program-reported, or calculated based on direct anthropometric assessment).
- Anthropometric measures post-discharge: Change in absolute height, weight, or mid-upper arm circumference (MUAC), change in anthropometric Z-scores, or velocity of child growth (i.e., grams/ week) up to 6 months post-discharge from treatment for growth faltering/failure, complicated or uncomplicated moderate or severe wasting, or oedema.

- All-cause mortality: Proportion of children who died due to any cause up to 6 months post-discharge from treatment for growth faltering/failure, complicated or uncomplicated moderate or severe wasting, or oedema based on caregiver or program report.
- Morbidity or recovery from co-morbidity: Proportion of children with and without morbidity at any point up to 6 months post-discharge from treatment for growth faltering/failure, complicated or uncomplicated moderate or severe wasting, or oedema based on caregiver or program report.

© 2023 Bliznashka L et al. *JAMA Network Open.*

eAppendix 3. Prespecified Subgroups for Subgroup Analyses

Child characteristics:

- Child age (0-6 months, 6-23 months, 24-59 months)
- Child sex (male/female)
- Type of growth failure/faltering at admission
- Acute malnutrition severity (moderate wasting, severe wasting, oedema) at admission
- Acute malnutrition phenotype (marasmus vs. kwashiorkor)
- Anthropometry at admission
- Anthropometry at discharge
- HIV status at admission
- Presence of co-morbidities at admission
- Author-defined categories of clinical risk

Study characteristics:

- Geographical location
- Type of context (humanitarian emergency vs. stable)
- Type of intervention setting (home- vs. community- vs. facility-based)
- Duration of intervention

© 2023 Bliznashka L et al. *JAMA Network Open.*

eAppendix 4. Effect of Antibiotic Prophylaxis With Co-trimoxazole

In the 0-6-month post-discharge follow-up period, Berkley et al. 2016 found that daily oral co-trimoxazole prophylaxis compared to placebo had no effect on the incidence of diarrhoea: incidence rate ratio (IRR) 1.21 (95% CI 0.85, 1.73) for inpatient treatment for diarrhoea post-discharge and IRR 1.11 (95% CI 0.92, 1.34) for outpatient treatment for diarrhoea post-discharge. Daily oral co-trimoxazole prophylaxis reduced the incidence of skin infections over 6 months of follow up by IRR 0.68 (95% CI 0.49-0.92). However, daily oral co-trimoxazole prophylaxis had no effect on morbidities requiring hospitalization within 6 months post-discharge: severe pneumonia (IRR 0.94, 95% CI 0.73, 1.23), sepsis (IRR 1.00, 95% CI 0.35, 2.86), malaria (IRR 0.56, 95% CI 0.15, 1.84), or other infections (IRR 1.05, 95% CI 0.77, 1.45). Daily oral co-trimoxazole prophylaxis also had no effect on outpatient morbidities within 6 months post-discharge: upper respiratory tract infections (IRR 0.98, 95% CI 0.86, 1.14), lower respiratory tract infections (IRR 0.90, 95% CI 0.77, 1.05), urinary tract infections (IRR 0.56, 95% CI 0.28, 1.09), or malaria (IRR 0.70, 95% CI 0.30, 1.63).[1]

At 12-months post-discharge from inpatient treatment for severe acute malnutrition (primary endpoint for the trial), daily oral co-trimoxazole prophylaxis had no effect on mortality or anthropometric recovery (**eTable 3**).

References

1. Berkley JA, Ngari M, Thitiri J, et al. Daily co-trimoxazole prophylaxis to prevent mortality in children with complicated severe acute malnutrition: A multicentre, double-blind, randomised placebo-controlled trial. *Lancet Glob Heal*. 2016;**4**(7):e464–e473.

© 2023 Bliznashka L et al. *JAMA Network Open.*

eAppendix 5. Effect of Psychosocial Stimulation Postdischarge From Inpatient Treatment

One study assessed the effect of psychosocial stimulation for children 6-60 months of age starting during in-patient treatment for uncomplicated SAM with individual and group sessions and continuing after discharge with at home visits.[2] This intervention had no effect on anthropometric Z-scores at 6 months post-discharge from inpatient treatment.[2] When the two RCTs providing psychosocial stimulation without food supplementation were pooled (where one assessed children at 6 months post-discharge from all nu*t*ritional treatment[3] and the other one assessed children at 6 months post-discharge from inpatient treatment[2]), results showed that psychosocial stimulation compared to routine care improved weight-for-age Z-score (mean difference (MD) 0.25, 95% CI 0.04, 0.46) and height-for-age Z-score (MD 0.24, 95% 0.04, 0.44) (**eTable 7**).

References

2. Abessa TG, Worku BN, Wondafrash M, et al. Effect of play-based family-centered psychomotor/psychosocial stimulation on the development of severely acutely malnourished children under six in a low-income setting: a randomized controlled trial. *BMC Pediatr*. 2019 Dec 14;**19**(1):336.
3. Nahar B, Hossain MI, Hamadani JD, et al. Effects of a community-based approach of food and psychosocial stimulation on growth and development of severely malnourished children in Bangladesh: A randomised trial. *Eur J Clin Nutr*. Nature Publishing Group; 2012;**66**(6):701–709.

© 2023 Bliznashka L et al. *JAMA Network Open.*

eFigure 1. Risk of Bias for Individually Randomized Clinical Studies Included in the Review

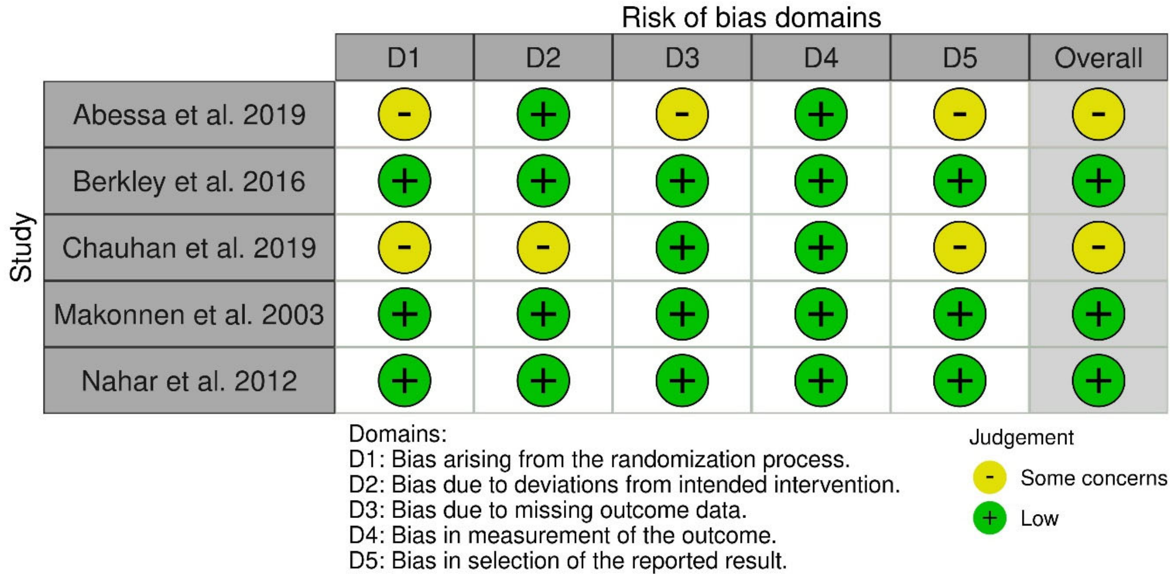

© 2023 Bliznashka L et al. *JAMA Network Open.*

eFigure 2. Risk of Bias for Cluster Randomized Clinical Studies Included in the Review

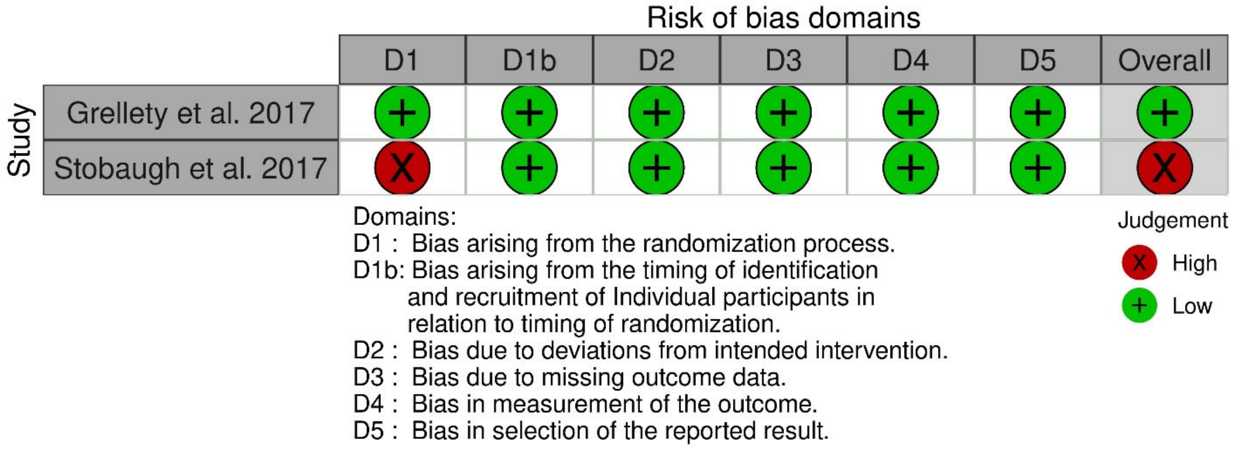

© 2023 Bliznashka L et al. *JAMA Network Open.*

eFigure 3. Risk of Bias for Observational Studies Included in the Review

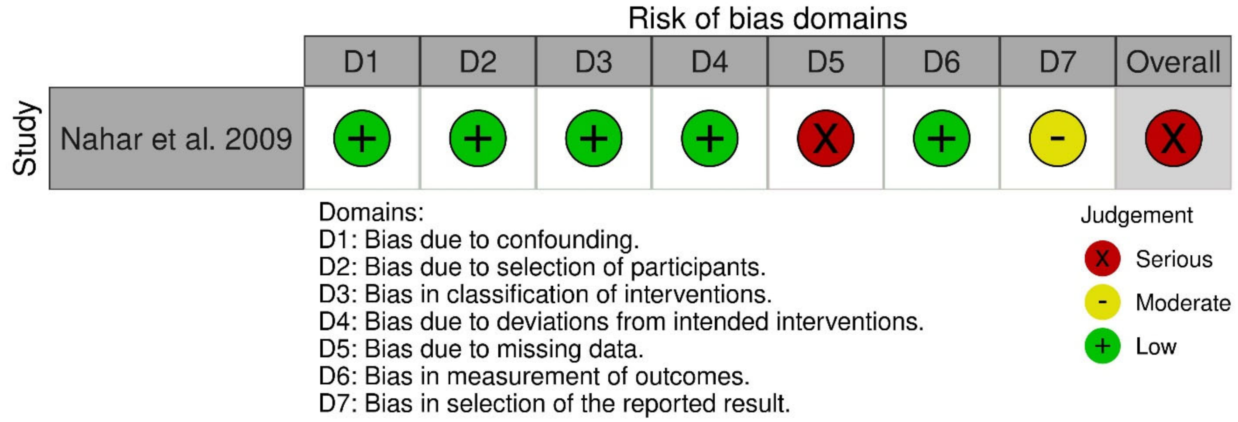

© 2023 Bliznashka L et al. *JAMA Network Open.*

eTable 1. Excluded Records During Full-Text Review and Reasons for Exclusion

Record	Author, year	Title	Reason for exclusion
1	Altman et al. 2018	The impact of adding a household wash package to CMAM program: A cluster-randomized controlled trial in Chad	No post-discharge intervention assessed
2	Alvear et al. 1991	Growth after early severe malnutrition	Ineligible comparator
3	Ashraf et al. 2012	A follow-up experience of 6 months after treatment of children with severe acute malnutrition in Dhaka, Bangladesh	No post-discharge intervention assessed
4	Chevalier et al. 1996	Immuno-nutritional recovery of children with severe malnutrition	No eligible outcomes
5	Daniel et al. 2021	A Mixed-Methods Cluster-Randomized Controlled Trial of a Hospital-Based Psychosocial Stimulation and Counseling Program for Caregivers and Children with Severe Acute Malnutrition	No post-discharge intervention assessed
6	Docey et al. 2018	Point-of-use water treatment improves recovery rates among children with severe acute malnutrition in Pakistan: results from a site-randomized trial	No post-discharge intervention assessed
7	Grantham-McGregor et al. 1980	Effect of long-term psychosocial stimulation on mental development of severely malnourished children	Ineligible comparator
8	Hother et al. 2016	Serum phosphate and magnesium in children recovering from severe acute undernutrition in Ethiopia: an observational study	No post-discharge intervention assessed
9	Khan et al. 2021	Comparison between different feeding protocols and existing protocol for the treatment of acute malnutrition (a	Ineligible study design – conference abstract

© 2023 Bliznashka L et al. *JAMA Network Open.*

Record	Author, year	Title	Reason for exclusion
		cluster randomized controlled clinical trial) (pilot study)	
10	Makonnen et al. 2003	A randomized controlled study of the impact of dietary zinc supplementation in the management of children with protein-energy malnutrition in Lesotho. II: Special investigations	No eligible outcomes
11	Mwangome et al. 2020	Individualized breastfeeding support for acutely ill, malnourished infants under 6 months old	No post-discharge intervention assessed
12	Mwangome et al. 2017	Diagnostic criteria for severe acute malnutrition among infants aged under 6 mo	No post-discharge intervention assessed
13	Nahar et al. 2015	Effect of a food supplementation and psychosocial stimulation trial for severely malnourished children on the level of maternal depressive symptoms in Bangladesh	No eligible outcomes
14	Njeru et al. 2021	Strengthening the role of community health workers in supporting the recovery of ill, undernourished children post hospital discharge: qualitative insights from key stakeholders in Bangladesh and Kenya	No post-discharge intervention assessed
15	Patel et al. 2010	Home-based rehabilitation of severely malnourished children in resource poor setting	No post-discharge intervention assessed
16	Patel et al. 2014	Evaluation of a malnutrition management program in Gujarat, India	Ineligible study design
17	Pietravalle at al. 2021	Nutritional education during rehabilitation of children 6-24 months with acute malnutrition, under unavailability of	No post-discharge intervention assessed

© 2023 Bliznashka L et al. *JAMA Network Open.*

Record	Author, year	Title	Reason for exclusion
		therapeutic/supplementary foods: a retrospective study in rural Angola	
18	Van Ryneveld et al. 2020	Mothers' experiences of exclusive breastfeeding in a postdischarge home setting	Ineligible study design

© 2023 Bliznashka L et al. *JAMA Network Open.*

eTable 2. Detailed Summary of Included Studies

Source	Population assessed	Intervention description	Comparator description	Intervention duration	Outcomes assessed	Findings
Abessa et al,[25] 2019	Children aged 6-60 mo with uncomplicated SAM, defined as WHZ <70% of median NCHS or MUAC <110 mm with length >65 mm or bilateral pitting edema	Psychosocial support stimulation: 2 phases of inpatient and outpatient of play-based psychosocial stimulation. The inpatient phase was provided at the hospital and included individual and group sessions in a playroom. The outpatient phase was provided at home and included 3 home visits over a period of 6 mo providing stimulation for the child and empowering the caregiver.	Children with uncomplicated SAM provided routine care which involved biomedical and dietary treatment	8-10 sessions during inpatient treatment, and up to 6 mo after discharge from inpatient treatment, including variable time during subsequent outpatient treatment and post-discharge from outpatient treatment	Anthropometric measures (HAZ, MUACZ, WAZ, WHZ)	The intervention did not improve anthropometric measures by end of follow-up
Berkley et al,[26] 2016	Children aged 60 d to 59 mo diagnosed with SAM (defined as MUAC <115 mm for children >6 mo and MUAC <110 mm for children 2-5 mos or kwashiorkor) who had completed the stabilization phase of treatment and had a negative HIV rapid antibody test	Biomedical: 6 mo of daily oral co-trimoxazole prophylaxis given as water-dispersible tablets of 120 mg/d for children aged <6 mo and 240 mg/d for children aged 6-59 mo	Placebo	6 mos starting at discharge from inpatient treatment (including variable time during subsequent outpatient treatment and postdischarge from outpatient treatment)	Relapse to SAM, sustained recovery, anthropometric measures (MUAC, WHZ, WAZ, HAZ, HCZ), all-cause mortality, morbidity or recovery from morbidity (pneumonia, severe pneumonia, diarrhea, severe diarrhea, malaria, skin or soft tissue infection, upper respiratory tract infection, lower respiratory tract infection, urinary tract infection)	The intervention did not reduce mortality or adverse events or improve anthropometric outcomes
Chauhan et al,[22] 2019	Children aged 6 mo to 5 y with SAM	Food supplementation: nonmilk based LTF provided at discharge and 3 follow-ups every two weeks. The nonmilk based LTF food contained wheat flour (30 g, 102 kcal, 3.63 g protein, 0.41 g fat), groundnuts (60 g, 342 kcal, 15.7 g protein, 23.8 g fat), sugar (30 g, 119 kcal, 0.03 g protein, 0 g fat) and oil (30 g, 270 kcal, 0 g protein, 30 g fat) for a total of 150 g, 833 kcal, 19.36 g protein and 54. 3 g	Advice on home-based diet, no LTF received	6 weeks starting at discharge from inpatient treatment (considered discharge from all nutritional treatment since there was no outpatient treatment program at the time the study was conducted)	Anthropometric measures (weight gain, MUAC, WHZ)	The intervention improved mean (SD) weight gain over 6 weeks of follow-up: 5.48 (0.55) g/kg/din the intervention group vs 2.47 (0.36) g/kg/d in the control group (*P* < .001). The intervention

© 2023 Bliznashka L et al. *JAMA Network Open*.

		fat. The LTF was provided in pouches. The amount of LTF was given according to child weight: 100-130 g/d for children weighing 3-4.9 kg; 200-250 g/d for children weighing 5-6.99 kg; 250-400 g/d for children weighing 7-9.9 kg; and 400-450 g/d for children weighing >10 kg.				reduced the proportion of children with MUAC <11.5 cm, increased the proportion of children with WHZ >2 SD, and increased the proportion of children labeled as cured
Grellety et al,[27] 2017	Children aged 6-59 mo in outpatient treatment for uncomplicated SAM, defined as MUAC <115 mm and/or WHZ <−3, and/or bilateral edema	Cash transfer: all caregivers with ≥1 children with SAM received an unconditional cash transfer of US $40 per month during treatment and follow-up for a total of 6 mo. The amount provided 70% of the monthly household income of very poor households.	Standard of care according to the national protocol for integrated management of acute malnutrition	A total of 6 mos starting at admission to outpatient treatment and continuing after discharge from nutritional treatment	Relapse (MAM relapse, SAM relapse), sustained recovery, anthropometric measures (height, weight, HAZ, WAZ, WHZ BMIZ, MUAC, MUACZ, MUAC-for-height *z* score)	During follow-up after discharge, the intervention decreased the risk of MAM and SAM relapse. Children in the intervention group continued to have higher weight and MUAC gain. Changes in WAZ, WHZ, BMIZ, MUACZ, and MUAC-for-height *z* score were all positive in the intervention group.
Makonnen et al,[23] 2003	Children aged 6 mo to 5 y with signs of protein energy malnutrition based on the 1999 Wellcome Classification or signs and symptoms of kwashiorkor	Biomedical: daily dose of 10 mg of zinc was administered from the first day of admission to 90 d postdischarge	Placebo	Starting at admission to inpatient treatment and continuing until 90 d after discharge from inpatient treatment (considered discharge from all nutritional treatment since there was no outpatient treatment program at the time the study was conducted)	Anthropometric measures (WAZ, MUAC), all-cause mortality, morbidity or recovery from morbidity (diarrhea, vomiting, fever, edema, acute respiratory tract infection, skin infection, pallor)	The intervention reduced morbidity at 30, 60 and 90 d postdischarge. The intervention improved WAZ at 30, 60 and 90 d post discharge and MUAC<5% at 60 and 90 d post discharge.
Nahar et al,[29] 2009	Children aged 6 to 24 mo hospitalized with SAM, defined as WAZ <50% of the median of the NCHS reference or WLZ <70% or nutritional edema	Psychosocial stimulation: in hospital, for the first 2 weeks, daily 0.5-hour group session and 0.5-hour individual play sessions. During 11 home visits, demonstration of developmentally appropriate play activities and provision of toys. On return to the hospital for follow-up visits, the intervention	Received routine nutritional and health care and health and nutrition education	Starting at admission to inpatient treatment and continuing for 6 mo after discharge from inpatient treatment (considered discharge from all nutritional treatment since	Anthropometric measures (WAZ, WHZ, HAZ)	The intervention improved WAZ at 6 mo postdischarge.

© 2023 Bliznashka L et al. *JAMA Network Open*.

		also received individual play sessions. A total of 18 sessions supervised sessions in hospital or at home.		there was no outpatient treatment program at the time the study was conducted)		
Nahar et al,[24] 2012	Children aged 6-24 mo hospitalized with severe underweight (defined as WAZ <−3 SD) without acute infections. Children with WHZ <−3 SD or edema were excluded from the study.	Psychosocial stimulation: individual play sessions and parental education for 1 h, including lending of toys and simple picture books, conducted at community clinics for 6 mo. Food supplementation: food packets for 3 mos: 1 per day for children aged 6-11 mo and 2 per day for children aged 12-24 mo containing 20 g of roasted rice powder, 10 g roasted lentil powder, 5 g molasses, and 3 g soya oil providing 150 kcal with 11% derived from proteins	Clinic control: fortnightly follow-up at clinic with growth monitoring, health education, and micronutrient supplementation Hospital control: Fortnightly follow up at the hospital with growth monitoring, health education, and micronutrient supplementation	6 mo Starting at discharge from inpatient treatment (considered discharge from all nutritional treatment since there was no outpatient treatment program at the time the study was conducted)	Anthropometric measures (HAZ, WAZ, WHZ)	No intervention effect on anthropometric outcomes after 6 mo of intervention. Any psychosocial stimulation improved WAZ compared with no16timulateon at 6 mo.

© 2023 Bliznashka L et al. *JAMA Network Open*.

Stobaugh et al,[28] 2017	Children aged 6-62 mos discharged as recovered from community-based treatment for MAM, defined as MUAC ≥125 mm	Intervention package consisting of the following 5 interventions. (1) food supplementation: 40 g/d of LNS that provided 200 kcal and 1 RDA of micronutrients for 8 weeks. The LNS consisted of 28% peanut paste, 18% nonfat dry skimmed milk powder, 24.5% palm oil, 21.2% sugar, 6.8% custom micronutrient mix, and 1.5% emulsifier. (2) Biomedical support: a single dose of albendazole (200 mg for children aged <2 y and 400 mg for children aged ≥2 y. (3) Biomedical support: zinc supplementation for 14 d consisting of 20 mg zinc sulfate starting at the time of discharge. (4) Malaria prevention: a single insecticide-treated bed net. (5) Malaria prevention: a monthly dose of 25 mg/kg of sulfadoxine- pyrimethamine for malaria chemoprophylaxis during the peak of the rainy season	Standard of care and routine nutrition and health counselling	Up to 1 y (depending on the individual intervention within the package) after discharge from outpatient treatment	MAM relapse, deterioration to severe wasting among children recovered from moderate wasting, sustained recovery, all-cause mortality	The intervention improved sustained recovery at 1, 3, and 6 mo post-discharge, but had no effect on sustained recovery at 12 mo (primary outcome). The intervention did not improve MAM relapse 1, 3, 6, or 12 mo post-discharge. The intervention did not improve deterioration to severe wasting at 1, 3, or 6 mo postdischarge, but had a positive association at 12 mo postdischarge. The intervention did not improve relapse-free survival at 1, 3, 6, or 12 mo postdischarge.

Abbreviations used: BMIZ, body mass index *z* score; HAZ; height/length-for-age *z* score; HCZ, head circumference-for-age *z* score; LNS, lipid-based nutrient supplement; LTF, local therapeutic food; MAM, moderate acute malnutrition; MUAC, mid–upper arm circumference; MUACZ, mid-upper arm circumference *z* score; NCHS, National Centre for Health Statistics; RDA, recommended daily allowance; SAM, severe acute malnutrition; WAZ, weight-for-age *z* score; WHZ; weight-for-height/length *z* score.

© 2023 Bliznashka L et al. *JAMA Network Open*.

eTable 3. GRADE Evidence Profile for the Effect of Daily Oral Co-trimoxazole Prophylaxis Compared With Routine Care (at 12 Months After Discharge From Inpatient Treatment)

Certainty assessment							№ of patients		Effect		Certainty
№ of studies	Study design	Risk of bias	Inconsistency	Indirectness	Imprecision	Other considerations	daily oral co-trimoxazole prophylaxis	placebo	Relative (95% CI)	Absolute (95% CI)	
**Mortality**											
1	randomised trials	not serious	not serious	not serious	serious[a]	none	122/887 (13.8%)	135/891 (15.2%)	**RR 0.91** (0.72 to 1.14)	**14 fewer per 1,000** (from 42 fewer to 21 more)	⊕⊕⊕◯ Moderate
**MUAC (cm) at 12 months**											
1	randomised trials	not serious	not serious	not serious	not serious	none	719	712	-	MD **0.1 lower** (0.24 lower to 0.04 higher)	⊕⊕⊕⊕ High
**WHZ at 12 months**											
1	randomised trials	not serious	not serious	not serious	not serious	none	719	712	-	MD **0.03 lower** (0.18 lower to 0.12 higher)	⊕⊕⊕⊕ High
**WAZ at 12 months**											
1	randomised trials	not serious	not serious	not serious	not serious	none	719	712	-	MD **0** (0.13 lower to 0.13 higher)	⊕⊕⊕⊕ High
**HAZ at 12 months**											
1	randomised trials	not serious	not serious	not serious	serious[b]	none	719	712	-	MD **0.07 higher** (0.073 lower to 0.21 higher)	⊕⊕⊕◯ Moderate
**Head circumference-for-age z-score**											

© 2023 Bliznashka L et al. *JAMA Network Open.*

Certainty assessment							№ of patients		Effect		Certainty
№ of studies	Study design	Risk of bias	Inconsistency	Indirectness	Imprecision	Other considerations	daily oral co-trimoxazole prophylaxis	placebo	Relative (95% CI)	Absolute (95% CI)	
1	randomised trials	not serious	not serious	not serious	not serious	none	719	712	-	MD **0.01 lower** (0.16 lower to 0.14 higher)	⨁⨁⨁⨁ High
**Anthropometric recovery (MUAC at least 12.5cm) at 12 months**											
1	randomised trials	not serious	not serious	not serious	not serious	none	602/887 (67.9%)	603/891 (67.7%)	**RR 1.00** (0.94 to 1.07)[c]	**0 fewer per 1,000** (from 41 fewer to 47 more)	⨁⨁⨁⨁ High
**Readmission**											
1	randomised trials	not serious	not serious	not serious	not serious[c]	none	There were 616 non-fatal admissions to hospital and 3266 non-fatal episodes of illness for which children were treated as outpatients. The incidence of readmission to hospital or death during follow-up was 57.1 per 100 child-years of observation (95% CI 54.6-59.6). We noted no significant differences in the overall rates of hospital admission or outpatient illness between intervention groups.				⨁⨁⨁⨁ High
**Relapse - not measured**											
-	-	-	-	-	-	-	-	-	-	-	-

CI: confidence interval; **MD:** mean difference; **RR:** risk ratio

Explanations
a. Serious imprecision: 95% CI around the absolute effect crosses the null and the effect ranges include an important appreciable differences from the point estimate for mortality.
b. Serious imprecision: 95% CI around the absolute effect crosses the null and the effect ranges include a trivial harm and small benefit.
c. There were no differences between arms in the proportion of children who had severe wasting or oedema at 12 months (RR 0.95, 95% CI: 0.60, 1.50) or moderate wasting at 12 months (RR 1.13, 95% CI: 0.83, 1.53).

© 2023 Bliznashka L et al. *JAMA Network Open*.

eTable 4. GRADE Evidence Profile for the Effect of Zinc Supplementation Compared With Placebo

Certainty assessment							№ of patients		Effect		Certainty
**№ of studies**	**Study design**	**Risk of bias**	**Inconsistency**	**Indirectness**	**Imprecision**	**Other considerations**	**Zinc supplementation**	**Placebo**	**Relative (95% CI)**	**Absolute (95% CI)**	
**Relapse - not measured**											
-	-	-	-	-	-	-	-	-	-	-	-
**Deterioration to severe wasting among children recovered from moderate wasting – not measured**											
-	-	-	-	-	-	-	-	-	-	-	-
**Readmission - not measured**											
-	-	-	-	-	-	-	-	-	-	-	-
**Sustained recovery - not measured**											
-	-	-	-	-	-	-	-	-	-	-	-
**Weight <60% of expected weight-for-age**											
1	randomised trials	not serious	not serious	not serious	serious[a]	none	5/138 (3.6%)	16/116 (13.8%)	**RR 0.26** (0.10 to 0.70)	**102 fewer per 1,000** (from 124 fewer to 41 fewer)	⊕⊕⊕◯ Moderate
**Mid-upper arm circumference percentile <5th**											
1	randomised trials	not serious	not serious	not serious	not serious	none	66/138 (47.8%)	81/116 (69.8%)	**RR 0.68** (0.55 to 0.85)	**223 fewer per 1,000** (from 314 fewer to 105 fewer)	⊕⊕⊕⊕ High
**All-cause mortality - not measured**											
-	-	-	-	-	-	-	-	-	-	-	
1	randomised trials	not serious	not serious	not serious	serious[a]	none	4/138 (2.9%)	31/116 (26.7%)	**RR 0.11** (0.04 to 0.30)	**238 fewer per 1,000** (from 257 fewer to 187 fewer)	

© 2023 Bliznashka L et al. *JAMA Network Open*.

Certainty assessment							№ of patients		Effect		Certainty
№ of studies	Study design	Risk of bias	Inconsistency	Indirectness	Imprecision	Other considerations	Zinc supplementation	Placebo	Relative (95% CI)	Absolute (95% CI)	
**Vomiting**											
1	randomised trials	not serious	not serious	not serious	serious[a]	none	1/138 (0.7%)	8/116 (6.9%)	**RR 0.11** (0.01 to 0.83)	**61 fewer per 1,000** (from 68 fewer to 12 fewer)	
**Fever**											
1	randomised trials	not serious	not serious	not serious	serious[a]	none	4/138 (2.9%)	12/116 (10.3%)	**RR 0.28** (0.09 to 0.85)	**74 fewer per 1,000** (from 94 fewer to 16 fewer)	
**Oedema**											
1	randomised trials	not serious	not serious	not serious	serious[a]	none	0/138 (0.0%)	0/116 (0.0%)	not estimable		
1	randomised trials	not serious	not serious	not serious	serious[a]	none	4/138 (2.9%)	45/116 (38.8%)	**RR 0.07** (0.03 to 0.20)	**361 fewer per 1,000** (from 376 fewer to 310 fewer)	
**Skin infection**											
1	randomised trials	not serious	not serious	not serious	serious[a]	none	1/138 (0.7%)	8/116 (6.9%)	**RR 0.11** (0.01 to 0.83)	**61 fewer per 1,000** (from 68 fewer to 12 fewer)	
**Pallor**											
1	randomised trials	not serious	not serious	not serious	not serious	none	32/138 (23.2%)	62/116 (53.4%)	**RR 0.43** (0.31 to 0.61)	**305 fewer per 1,000** (from 369 fewer to 208 fewer)	

CI: confidence interval; **RR:** risk ratio

© 2023 Bliznashka L et al. *JAMA Network Open*.

Explanations

a. Downgraded due to small sample size, few events, and wide confidence intervals.

© 2023 Bliznashka L et al. *JAMA Network Open*.

eTable 5. GRADE Evidence Profile for the Effect of Food Supplementation Compared With Routine Care

Certainty assessment							№ of patients		Effect		Certainty
**№ of studies**	**Study design**	**Risk of bias**	**Inconsistency**	**Indirectness**	**Imprecision**	**Other considerations**	**Nutrition support**	**Routine care**	**Relative (95% CI)**	**Absolute (95% CI)**	
**Relapse – not measured**											
-	-	-	-	-	-	-	-	-	-	-	-
**Deterioration to severe wasting among children recovered from moderate wasting – not measured**											
-	-	-	-	-	-	-	-	-	-	-	-
**Readmission – not measured**											
-	-	-	-	-	-	-	-	-	-	-	-
**Sustained recovery – not measured**											
-	-	-	-	-	-	-	-	-	-	-	-
**Height-for-age Z-score**											
1	randomised trials	not serious	not serious	not serious	serious[a]	none	101	201	-	MD **0.00 higher** (0.38 lower to 0.38 higher)	⨁⨁⨁◯ Moderate
**Weight-for-age Z-score**											
1	randomised trials	not serious	not serious	not serious	serious[a]	none	101	201	-	MD **0.10 lower** (0.39 lower to 0.19 higher)	⨁⨁⨁◯ Moderate
**Weight-for-height Z-score**											
2	randomised trials	not serious	not serious	not serious	serious[a]	none	141	241	-	Not estimated[c]	⨁⨁⨁◯ Moderate

© 2023 Bliznashka L et al. *JAMA Network Open.*

Certainty assessment							№ of patients		Effect		Certainty
№ of studies	Study design	Risk of bias	Inconsistency	Indirectness	Imprecision	Other considerations	Nutrition support	Routine care	Relative (95% CI)	Absolute (95% CI)	
**Weight gain (g/kg/day)**											
1	randomised trials	serious	not serious	not serious	serious[a]	none	40	40	-	MD **3.01 higher** (2.81 higher to 3.21 higher)	⨁⨁◯◯ Low
**Mid-upper arm circumference**											
1	randomised trials	serious	not serious	not serious	serious[b]	none	-/40	-/40	Not estimable[d]	-	⨁⨁◯◯ Low
**All-cause mortality – not measured**											
-	-	-	-	-	-	-	-	-	-	-	-
**Morbidity or recovery from co-morbidity – not measured**											
-	-	-	-	-	-	-	-	-	-	-	-

CI: confidence interval; **MD:** mean difference

Explanations

a. Downgraded due to small sample size and wide confidence intervals.
b. Downgraded due to small sample size and no measure of uncertainty reported.
c. Not estimated: One study categorised weight-for-height (<-3 SD, -3 SD to -2 SD, and >-2 SD) and did not provide estimate for continuous weight-for-height Z-score. Therefore, we could not pool the two studies.
d. Not estimable: This study categorised mid-upper arm circumference (<11.5 cm, 11.5-12.5 cm, and >12.5 cm) and did not provide number of observations by category. Therefore, we could not estimate relative risk.

© 2023 Bliznashka L et al. *JAMA Network Open.*

eTable 6. GRADE Evidence Profile for the Effect of Psychosocial Stimulation Compared With Routine Care (at 6 Months After Discharge From All Nutritional Treatment)

Certainty assessment							№ of patients		Effect		Certainty
№ of studies	Study design	Risk of bias	Inconsistency	Indirectness	Imprecision	Other considerations	Psychosocial support	routine care	Relative (95% CI)	Absolute (95% CI)	
**Relapse – not measured**											
-	-	-	-	-	-	-	-	-	-	-	-
**Deterioration to severe wasting among children recovered from moderate wasting – not measured**											
-	-	-	-	-	-	-	-	-	-	-	-
**Readmission – not measured**											
-	-	-	-	-	-	-	-	-	-	-	-
**Sustained recovery – not measured**											
-	-	-	-	-	-	-	-	-	-	-	-
**Height-for-age Z-score**											
1	randomised trial	serious	serious[a]	not serious	serious[b]	none	102	201	-	MD **0.20 higher** (0.14 lower to 0.54 higher) to MD 0.40 **higher** (0.06 higher to 0.74 higher)	⨁◯◯◯ Very low
**Weight-for-age Z-score**											

© 2023 Bliznashka L et al. *JAMA Network Open.*

Certainty assessment							№ of patients		Effect		Certainty
№ of studies	Study design	Risk of bias	Inconsistency	Indirectness	Imprecision	Other considerations	Psychosocial support	routine care	Relative (95% CI)	Absolute (95% CI)	
1	randomised trial	serious	serious[a]	not serious	serious[b]	none	102	201	-	MD **0.30 higher** (0.07 lower to 0.67 higher) to MD 0.20 **higher** (0.22 lower to 0.62 higher)	⨁◯◯◯ Very low
**Weight-for-age Z-score**											
1	observational studies	serious	not serious	not serious	serious[b]	none	54	43	-	MD **0.5 higher** (0.01 higher to 0.99 higher)	⨁◯◯◯ Very low
**Weight-for-height Z-score**											
1	randomised trial	serious	serious[a]	not serious	serious[b]	none	102	201	-	MD **0.10 higher** (0.26 lower to 0.46 higher) to MD 0.10 lower (0.46 lower to 0.26 higher)	⨁◯◯◯ Very low
**Mid-upper arm circumference Z-score – not measured**											
-	-	-	-	-	-	-	-	-	-	-	-
**All-cause mortality – not measured**											
-	-	-	-	-	-	-	-	-	-	-	-

© 2023 Bliznashka L et al. *JAMA Network Open*.

Certainty assessment							№ of patients		Effect		Certainty
№ of studies	Study design	Risk of bias	Inconsistency	Indirectness	Imprecision	Other considerations	Psychosocial support	routine care	Relative (95% CI)	Absolute (95% CI)	
**Morbidity or recovery from co-morbidity – not measured**											
-	-	-	-	-	-	-	-	-	-	-	-

CI: confidence interval; **MD:** mean difference

Explanations

a. Downgraded due to methodological inconsistencies (e.g., intervention intensity, modality, duration)
b. Downgraded due to small sample sizes and wide confidence intervals.

© 2023 Bliznashka L et al. *JAMA Network Open.*

eTable 7. GRADE Evidence Profile for the Effect of Psychosocial Stimulation Compared With Routine Care (at 6 Months After Discharge From Inpatient Treatment)

Certainty assessment							№ of patients		Effect		Certainty
№ of studies	Study design	Risk of bias	Inconsistency	Indirectness	Imprecision	Other considerations	psychosocial stimulation	routine care	Relative (95% CI)	Absolute (95% CI)	
**Mortality - not measured**											
-	-	-	-	-	-	-	-	-	-	-	-
**WHZ (follow-up: 6 months)**											
2	randomised trials	serious[a]	not serious	not serious	serious[b]	none	240	293	-	MD **0.02 higher** (0.15 lower to 0.2 higher)	⊕⊕◯◯ Low
**WAZ (follow-up: 6 months)**											
2	randomised trials	serious[a]	not serious	not serious	serious[c]	none	240	293	-	MD **0.25 higher** (0.04 higher to 0.46 higher)	⊕⊕◯◯ Low
**HAZ (follow-up: 6 months)**											
2	randomised trials	serious[a]	not serious	not serious	serious[c]	none	240	293	-	MD **0.24 higher** (0.04 higher to 0.44 higher)	⊕⊕◯◯ Low
**Sustained recovery - not measured**											
-	-	-	-	-	-	-	-	-	-	-	-
**Readmission - not measured**											
-	-	-	-	-	-	-	-	-	-	-	-
**Relapse - not measured**											

© 2023 Bliznashka L et al. *JAMA Network Open.*

Certainty assessment							№ of patients		Effect		Certainty
**№ of studies**	**Study design**	**Risk of bias**	**Inconsistency**	**Indirectness**	**Imprecision**	**Other considerations**	**psychosocial stimulation**	**routine care**	**Relative (95% CI)**	**Absolute (95% CI)**	
-	-	-	-	-	-	-	-	-	-	-	-
**Deterioration to severe wasting - not measured**											
-	-	-	-	-	-	-	-	-	-	-	-
**Child development (follow-up: 6 months)**											
2	randomised trials	serious[a]	not serious	not serious	serious[d]	none	Authors of the Nahar 2012 study created a combined PS group (with or without food supplementation) and compared to the other three groups (FS and controls). The combined PS group had higher mental development scores (mental development index, based on the BSID) (regression coefficient = 5.4, P=0.017, 95% CI: 1 - 9.9). There was no effect of PS on psychomotor development. The Abessa 2019 study showed that PS had higher gross motor scores (0.88 points higher, P< 0.001) and fine motor scores (1.09 points higher, P=0.001) based on the Denver II.				⨁⨁◯◯ Low

CI: confidence interval; **MD:** mean difference

Explanations

a. Serious risk of bias: One of the two studies (Abessa et al. 2019) had some concerns (for randomisation, missing outcome data and reporting) while the other study (Nahar et al. 2012) had low risk of bias.
b. Serious imprecision: 95% CI around the absolute effect does cross the null, and effect ranges from a trivial harm to small benefit.
c. Serious imprecision: 95% CI around the absolute effect does not cross the null, however the effect ranges from a trivial benefit to appreciable benefit.
d. Serious imprecision: Unclear descriptions or estimates provided for effects and some concerns around small sample size.

© 2023 Bliznashka L et al. *JAMA Network Open.*

eTable 8. GRADE Evidence Profile for the Effect of Unconditional Cash Transfers Compared With Routine Care

Certainty assessment							№ of patients		Effect		Certainty
№ of studies	Study design	Risk of bias	Inconsistency	Indirectness	Imprecision	Other considerations	Unconditional cash transfers	Routine care	Relative (95% CI)	Absolute (95% CI)	
**Relapse to moderate acute malnutrition**											
1	randomised trials	not serious	not serious	serious[a]	not serious[a]	none	91/707 (12.9%)	292/660 (44.2%)	**RR 0.29** (0.24 to 0.36)	**314 fewer per 1,000** (from 336 fewer to 283 fewer)	⊕⊕⊕◯ Moderate
**Relapse to severe acute malnutrition**											
1	randomised trials	not serious	not serious	serious[a]	not serious[a]	none	24/707 (3.4%)	73/660 (11.1%)	**RR 0.34** (0.22 to 0.53)	**73 fewer per 1,000** (from 86 fewer to 52 fewer)	⊕⊕⊕◯ Moderate
**Deterioration to severe wasting among children recovered from moderate wasting – not measured**											
-	-	-	-	-	-	-	-	-	-	-	-
**Readmission – not measured**											
-	-	-	-	-	-	-	-	-	-	-	-
**Sustained recovery – not measured**											
-	-	-	-	-	-	-	-	-	-	-	-
**Height change (mm/week)**											
1	randomised trials	not serious	not serious	not serious	serious[a]	none	707	660	-	MD **0.10 lower** (0.20 lower to 0.00)	⊕⊕⊕◯ Moderate
**Weight change (g/kg/day)**											
1	randomised trials	not serious	not serious	not serious	serious[a]	none	707	660	-	MD **0.40 higher** (0.33 higher to 0.47 higher)	⊕⊕⊕◯ Moderate
**Mid-upper arm circumference change (mm/day)**											

© 2023 Bliznashka L et al. *JAMA Network Open.*

Certainty assessment							№ of patients		Effect		Certainty
№ of studies	Study design	Risk of bias	Inconsistency	Indirectness	Imprecision	Other considerations	Unconditional cash transfers	Routine care	Relative (95% CI)	Absolute (95% CI)	
1	randomised trials	not serious	not serious	not serious	serious[a]	none	707	660	-	MD **0.05 higher** (0.04 higher to 0.05 higher)	⊕⊕⊕◯ Moderate
**Height-for-age Z-score change (Z-score/month)**											
1	randomised trials	not serious	not serious	not serious	serious[a]	none	707	660	-	MD **0.02 lower** (0.04 lower to 0.00)	⊕⊕⊕◯ Moderate
**Weight-for-age Z-score change (Z-score/month)**											
1	randomised trials	not serious	not serious	not serious	serious[a]	none	707	660	-	MD **0.09 higher** (0.07 higher to 0.11 higher)	⊕⊕⊕◯ Moderate
**Weight-for-height Z-score change (Z-score/month)**											
1	randomised trials	not serious	not serious	not serious	serious[a]	none	707	660	-	MD **0.16 higher** (0.14 higher to 0.18 higher)	⊕⊕⊕◯ Moderate
**Body mass index Z-score change (Z-score/month)**											
1	randomised trials	not serious	not serious	not serious	serious[a]	none	707	660	-	MD **0.16 higher** (0.13 higher to 0.19 higher)	⊕⊕⊕◯ Moderate
**Mid-upper arm circumference Z-score change (Z-score/month)**											
1	randomised trials	not serious	not serious	not serious	serious[a]	none	707	660	-	MD **0.13 higher** (0.12 higher to 0.15 higher)	⊕⊕⊕◯ Moderate

© 2023 Bliznashka L et al. *JAMA Network Open.*

Certainty assessment							№ of patients		Effect		Certainty
**№ of studies**	**Study design**	**Risk of bias**	**Inconsistency**	**Indirectness**	**Imprecision**	**Other considerations**	**Unconditional cash transfers**	**Routine care**	**Relative (95% CI)**	**Absolute (95% CI)**	
**Mid-upper arm circumference for height Z-score change (Z-score/month)**											
1	randomised trials	not serious	not serious	not serious	serious[a]	none	707	660	-	MD **0.14 higher** (0.12 higher to 0.15 higher)	⊕⊕⊕◯ Moderate
**All-cause mortality – not measured**											
-	-	-	-	-	-	-	-	-	-	-	-
**Morbidity or recovery from co-morbidity – not measured**											
-	-	-	-	-	-	-	-	-	-	-	-

CI: confidence interval; **HR:** hazard Ratio; **MD:** mean difference

Explanations

a. Downgraded due to single trial with limited generalizability across contexts.

© 2023 Bliznashka L et al. *JAMA Network Open.*

eTable 9. GRADE Evidence Profile for the Effect of an Integrated Package of Medical Care, Food Supplementation, and Malaria Prevention Compared With Routine Care

Certainty assessment							№ of patients		Effect		Certainty
**№ of studies**	**Study design**	**Risk of bias**	**Inconsistency**	**Indirectness**	**Imprecision**	**Other considerations**	**Nutrition support**	**Routine care**	**Relative (95% CI)**	**Absolute (95% CI)**	
**Relapse to moderate acute malnutrition**											
1	randomised trials	very serious[a]	not serious	not serious	serious[b]	none	230/769 (29.9%)	234/718 (32.6%)	**RR 0.92** (0.79 to 1.07)	**26 fewer per 1,000** (from 68 fewer to 23 more)	⊕◯◯◯ Very low
**Deterioration to severe wasting among children recovered from moderate wasting**											
1	randomised trials	very serious[a]	not serious	not serious	not serious	none	18/769 (2.3%)	24/718 (3.3%)	**RR 0.70** (0.38 to 1.28)	**10 fewer per 1,000** (from 21 fewer to 9 more)	⊕⊕◯◯ Low
**Readmission - not measured**											
-	-	-	-	-	-	-	-	-	**-**	**-**	-
**Sustained recovery**											
1	randomised trials	very serious[a]	not serious	not serious	serious[c]	none	491/769 (63.8%)	421/718 (58.6%)	**RR 1.09** (1.00 to 1.18)	**53 more per 1,000** (from 0 fewer to 106 more)	⊕◯◯◯ Very low
**Anthropometric measures - not measured**											
-	-	-	-	-	-	-	-	-	**-**	**-**	-
**All-cause mortality**											

© 2023 Bliznashka L et al. *JAMA Network Open*.

Certainty assessment							№ of patients		Effect		Certainty
**№ of studies**	**Study design**	**Risk of bias**	**Inconsistency**	**Indirectness**	**Imprecision**	**Other considerations**	**Nutrition support**	**Routine care**	**Relative (95% CI)**	**Absolute (95% CI)**	
1	randomised trials	very serious[a]	not serious	not serious	serious[d]	none	7/769 (0.9%)	2/718 (0.3%)	**RR 3.27** (0.68 to 15.68)	**6 more per 1,000** (from 1 fewer to 41 more)	⊕◯◯◯ Very low
**Morbidity or recovery from co-morbidity - not measured**											
-	-	-	-	-	-	-	-	-	-	-	-

CI: confidence interval; **RR:** risk ratio

Explanations

a. Downgraded due to no random allocation or allocation concealment.
b. Downgraded due to: 95% CI around the absolute effect does cross the null, and effect ranges from a small benefit to trivial harm.
c. Downgraded due to: 95% CI around the absolute effect does cross the null, and effect ranges from a no effect to moderate benefit.
d. Downgraded due to: 95% CI around the absolute effect crosses the null and the effect ranges include an important appreciable difference (harm) from the point estimate for mortality.

© 2023 Bliznashka L et al. *JAMA Network Open.*
