# Supplementary material for: Analysis of Postdischarge Interventions for Children Treated for Moderate or Severe Wasting, Growth Faltering or Failure, or Edema: A Systematic Review
Source: JAMA Netw Open. 2023 May 24;6(5):e2315077. doi: 10.1001/jamanetworkopen.2023.15077 (PMC10209742; doi:10.1001/jamanetworkopen.2023.15077)
Supplement: Supplement 2. — Data Sharing Statement [file jamanetwopen-e2315077-s002.pdf]

Data Sharing Statement

Bliznashka. Analysis of Postdischarge Interventions for Children Treated for Moderate or Severe Wasting, Growth Faltering or Failure, or Edema. *JAMA Netw Open*. Published May 24, 2023. doi:10.1001/jamanetworkopen.2023.15077

Data
Data available: Yes
Data types: Other (please specify)
Additional Information: All data are included in the manuscript and appendix.
How to access data: All data are included in the manuscript and appendix.
When available: With publication

Supporting Documents
Document types: None

Additional Information
Who can access the data: N/A
Types of analyses: N/A
Mechanisms of data availability: N/A
